# National Black HIV/AIDS Awareness Day — February 7, 2018

**DOI:** 10.15585/mmwr.mm6704a1

**Published:** 2018-02-02

National Black HIV/AIDS Awareness Day is observed each year on February 7 to emphasize the continuing disproportionate impact of human immunodeficiency virus (HIV) infection and acquired immunodeficiency syndrome (AIDS) on the U.S. black/African American (black) population.

In 2014, non-Hispanic blacks represented 12% of the U.S. population (*1*) and the estimated 471,500 blacks living with diagnosed and undiagnosed HIV infection accounted for 43% of all persons living with diagnosed and undiagnosed HIV (*2*). In 2016, blacks represented 12% of the U.S. population (*1*), and blacks with new HIV diagnoses accounted for 44% of all new HIV diagnoses (https://www.cdc.gov/hiv/pdf/library/reports/surveillance/cdc-hiv-surveillance-report-2016-vol-28.pdf).

In 2014, among blacks living with diagnosed HIV infection, in 38 jurisdictions with complete reporting of CD4 and viral load data, 69.8% received HIV medical care, and 51.5% were virally suppressed (viral load test of <200 copies of HIV RNA/mL) (*2*). A study reported in this issue of *MMWR* found racial and ethnic disparities in viral suppression and transmission risk (*3*).

CDC supports a range of efforts to reduce the risk for acquiring or transmitting HIV infection among blacks. Additional information is available at https://www.cdc.gov/features/BlackHIVAIDSAwareness.
